# Supplementary material for: Managing Microbiota Activity of Apis mellifera with Probiotic (Bactocell®) and Antimicrobial (Fumidil B®) Treatments: Effects on Spring Colony Strength
Source: Microorganisms. 2024 Jun 6;12(6):1154. doi: 10.3390/microorganisms12061154 (PMC11205764; doi:10.3390/microorganisms12061154)
Supplement: Supplementary file 1 [file microorganisms-12-01154-s001.zip › microorganisms-3016785-supplementary.pdf]

- **Group homogeneity at t0:**

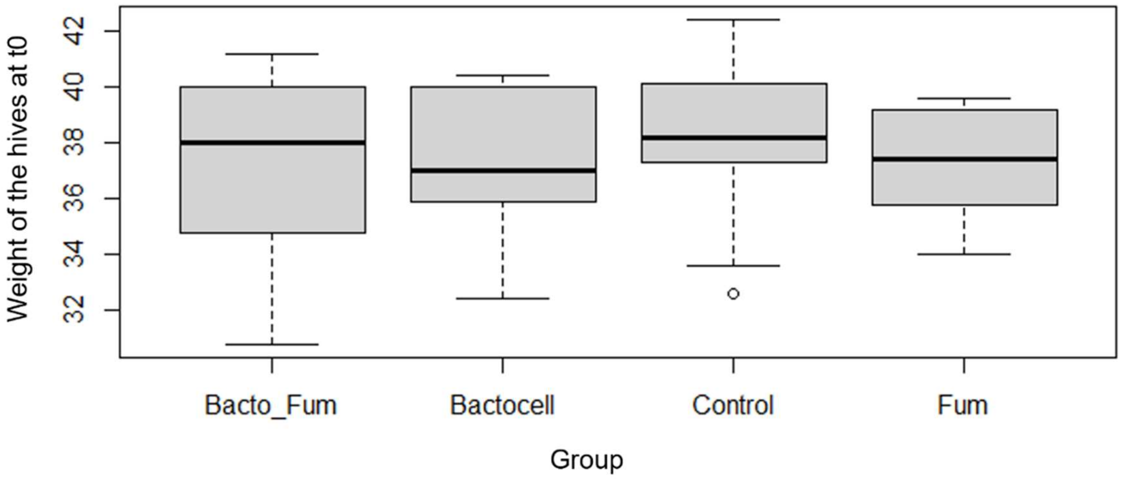

Figure S5. Hive weight by group at t0 (before treatment). Groups are homogeneous.

Group homogeneity at t0:

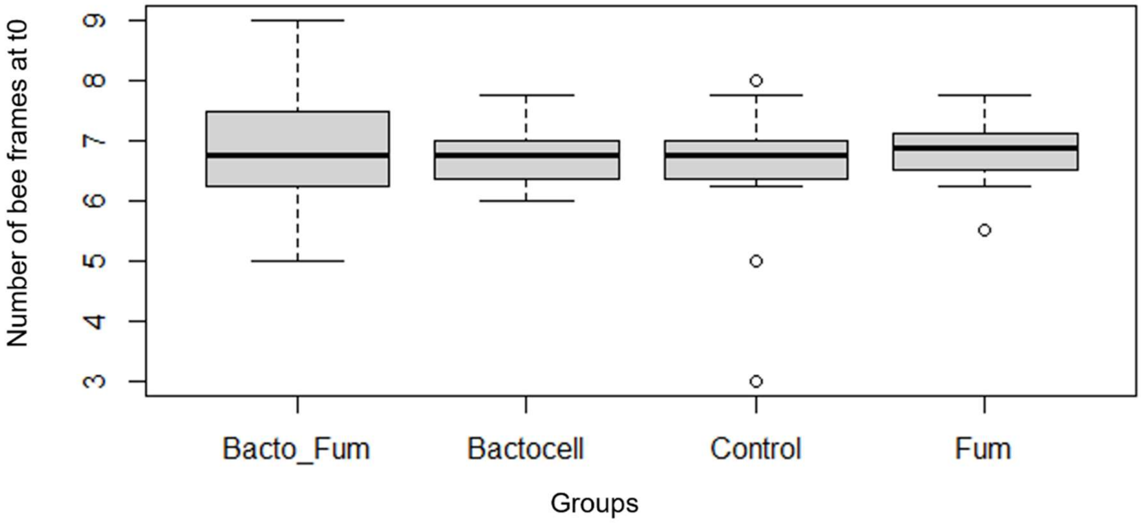

Figure S6. Number of bee frame per hive by group at t0 (before treatment). Groups are homogeneous.

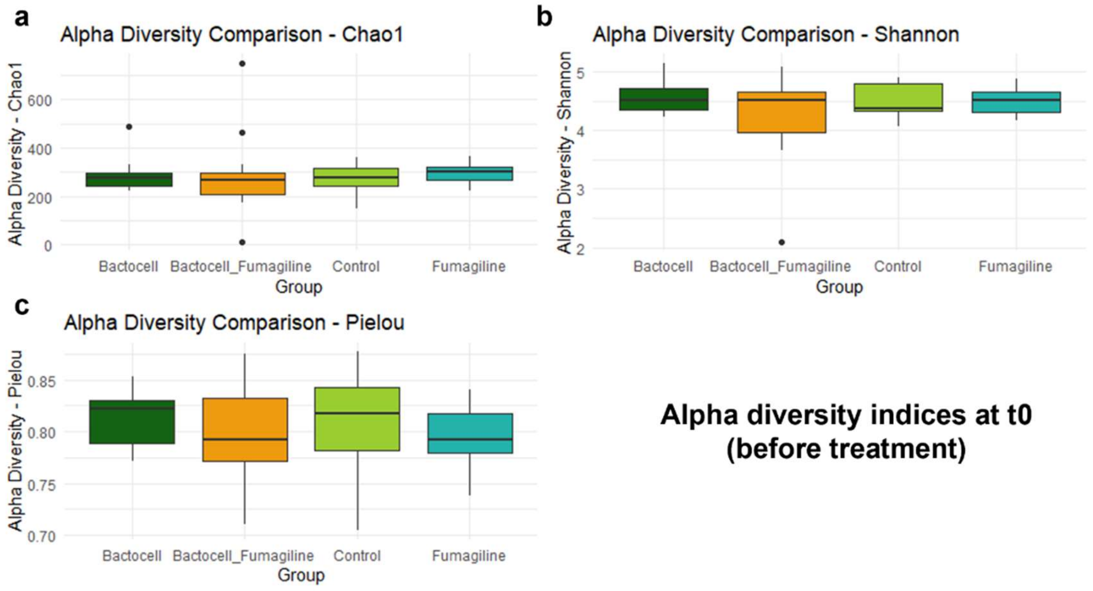

Figure S7a. boxplot representing the alpha diversity of bee microbiota from the colonies according to the group and at t0 (before treatment). **a**.Chao1 indice (specific richness). **b**.Shannon indice (diversity and equitability). **c**. Pielou indice (equitability)

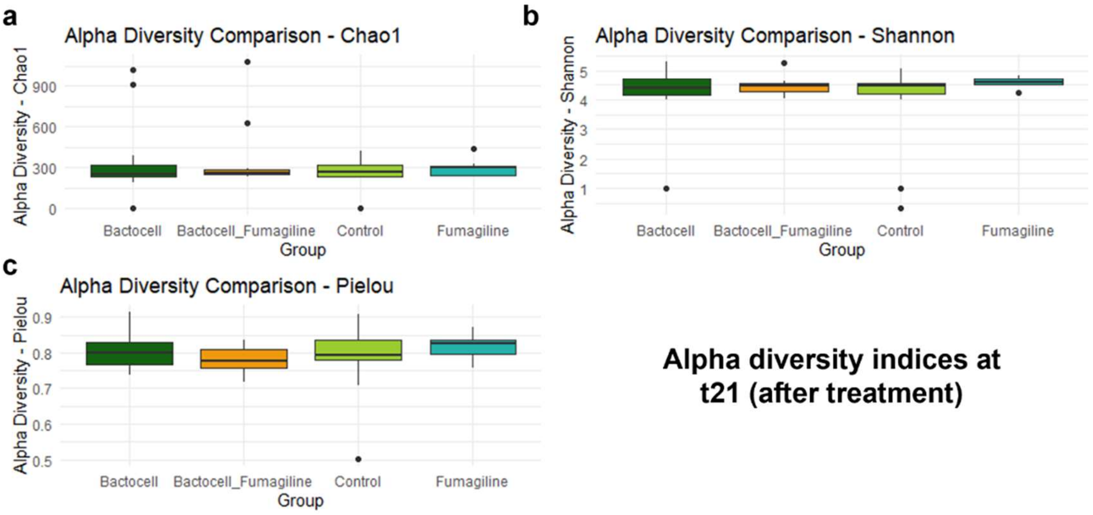

Figure S7b. boxplot representing the alpha diversity of bee microbiota from the colonies according to the group and at t21 (after treatment). **a**.Chao1 indice (specific richness). **B**.Shannon indice (diversity and equitability). **C**. Pielou indice (equitability)

Figure S8a

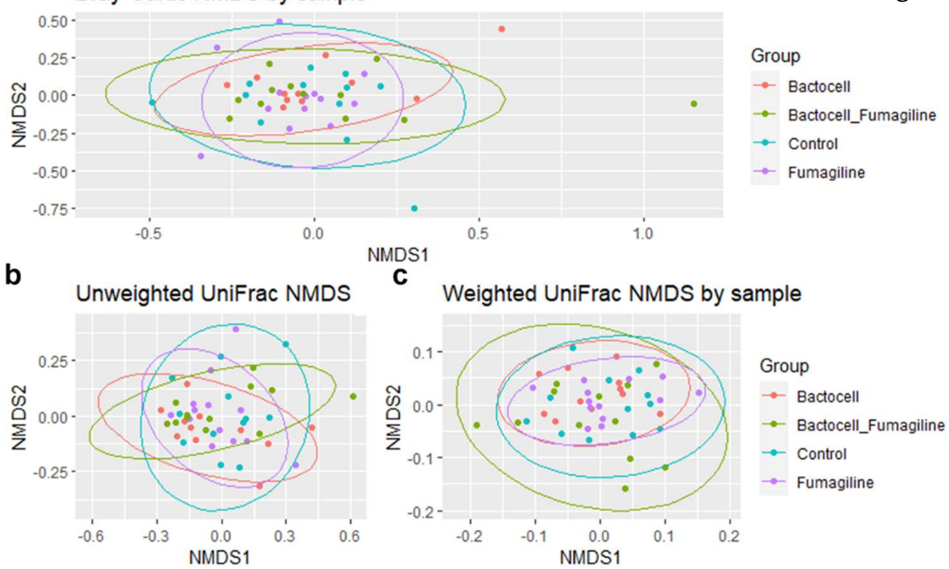

Figure S8a. Non-metric Multidimensional Scaling (NMDS) analysis of the microbiota of the bees from the colonies tested, colored by groups and at t0. **A**. Bray-Curtis distances**. b**. Unifrac distances. **c**. Weighted Unifrac distances.

Figure S8b

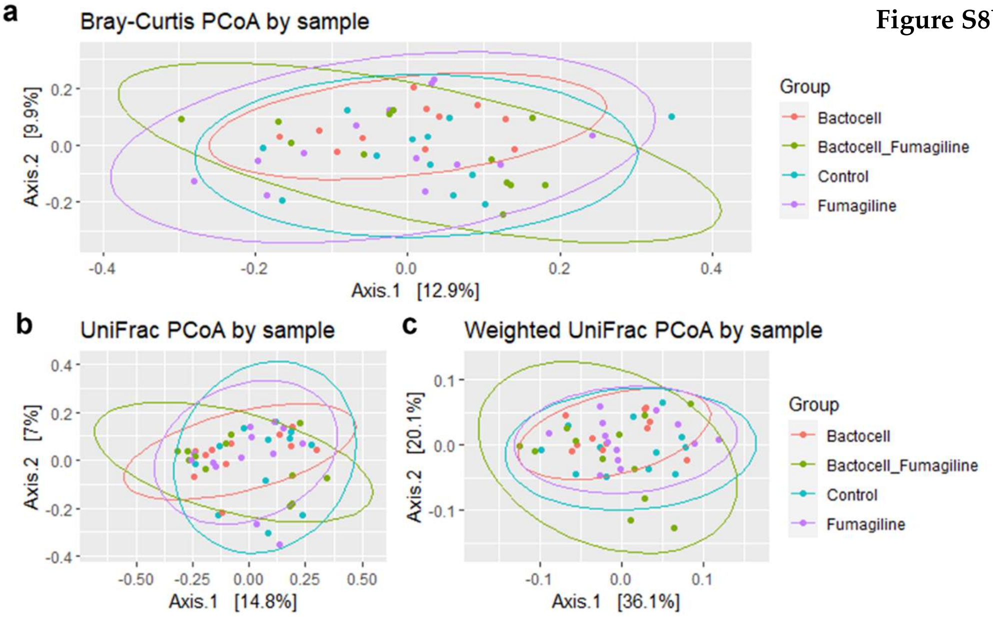

Figure S8b. Principal Component Analysis (PcoA) of the microbiota of the bees from the colonies tested, colored by groups and at t0. **A**. Bray-Curtis distances**. b**. Unifrac distances. **c**. Weighted Unifrac distances.

Figure S8c

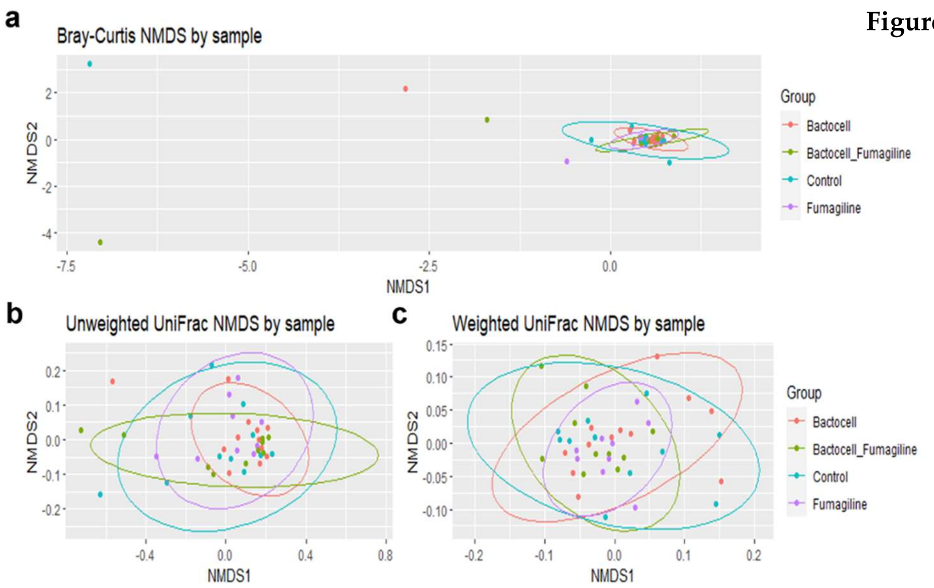

Figure S8c. Non-metric Multidimensional Scaling (NMDS) analysis of the microbiota of the bees from the colonies tested, colored by groups and at t21. **A**. Bray-Curtis distances**. b**. Unifrac distances. **c**. Weighted Unifrac distances.

Figure S8d

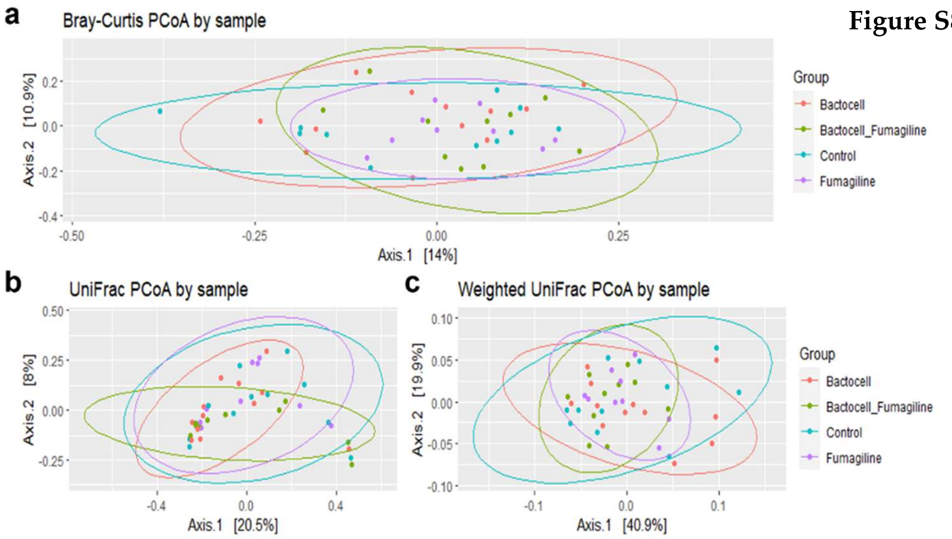

Figure S8d. Principal Component Analysis (PcoA) of the microbiota of the bees from the colonies tested, colored by groups and at t21. **A**. Bray-Curtis distances**. b**. Unifrac distances. **c**. Weighted Unifrac distances.

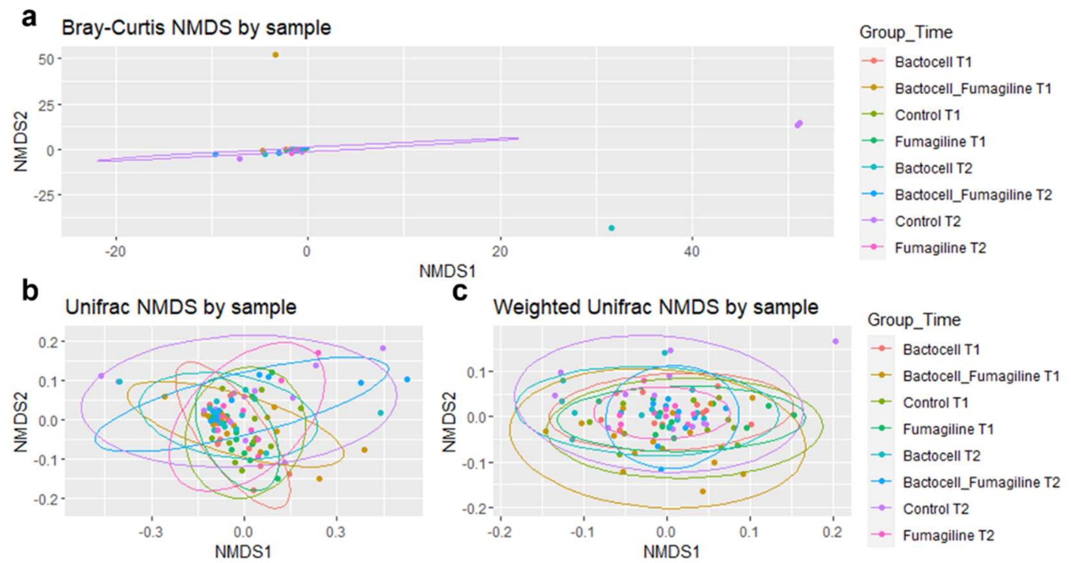

Figure S8e. Non-metric Multidimensional Scaling (NMDS) analysis of the microbiota of the bees from the colonies tested, colored by groups and time, for t0 and t21. **A**. Bray-Curtis distances**. b**. Unifrac distances. **c**. Weighted Unifrac distances.

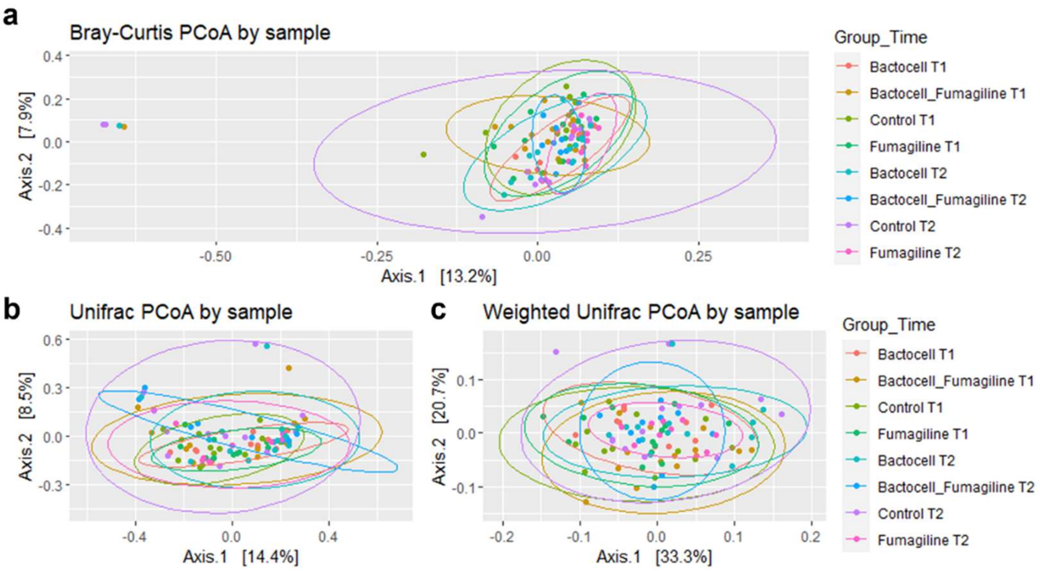

Figure S8f. Principal Component Analysis (PcoA) of the microbiota of the bees from the colonies tested, colored by groups and time, for t0 and t21. **A**. Bray-Curtis distances**. b**. Unifrac distances. **c**. Weighted Unifrac distances.

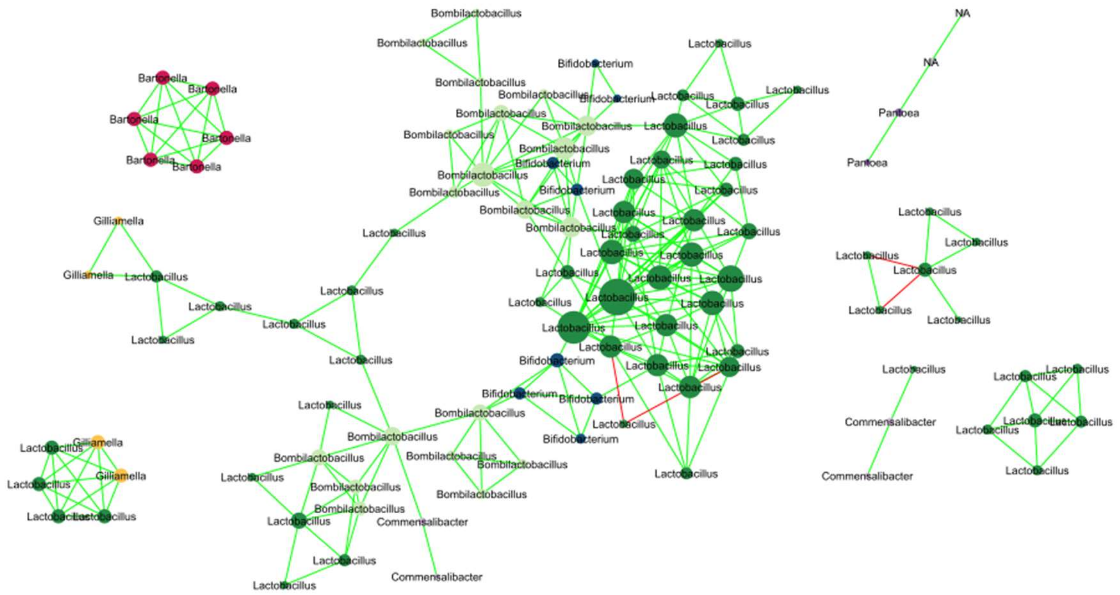

Figure S9a. Network interaction of the bees sampled from the control group at t0

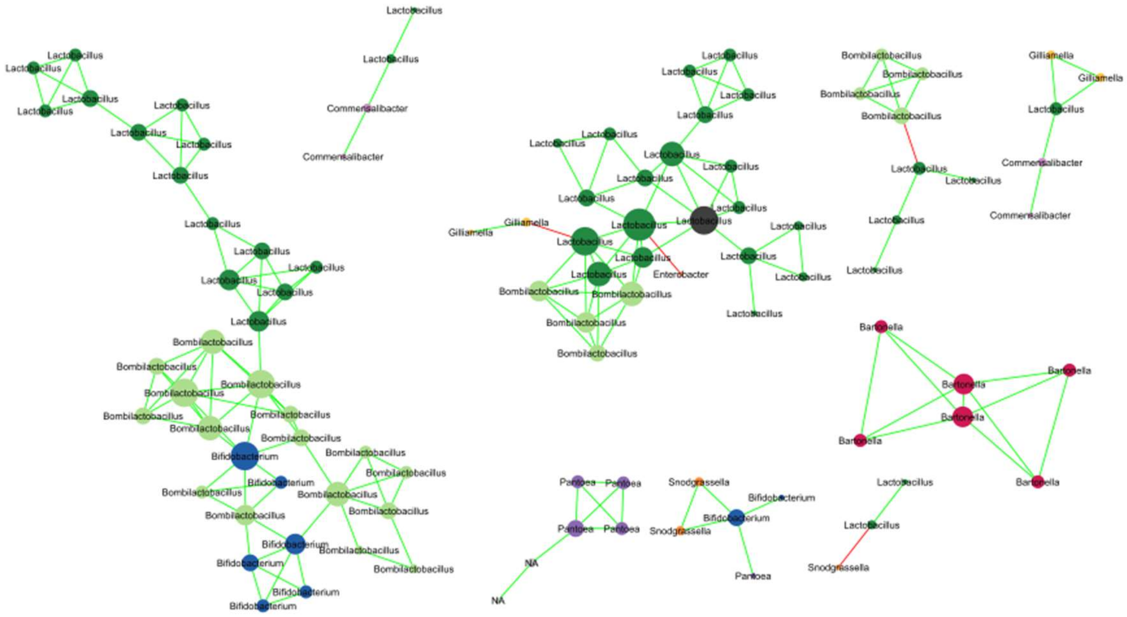

Figure S9b. Network interaction of the bees sampled from the Bactocell® group at t0

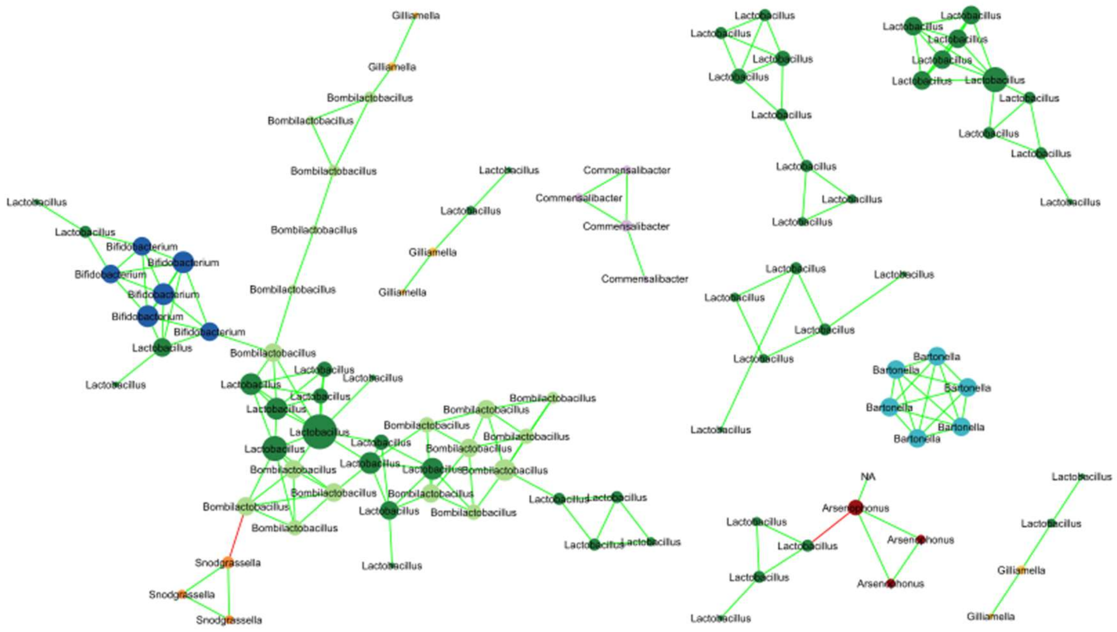

Figure S9c. Network interaction of the bees sampled from the Fumagiline group at t0

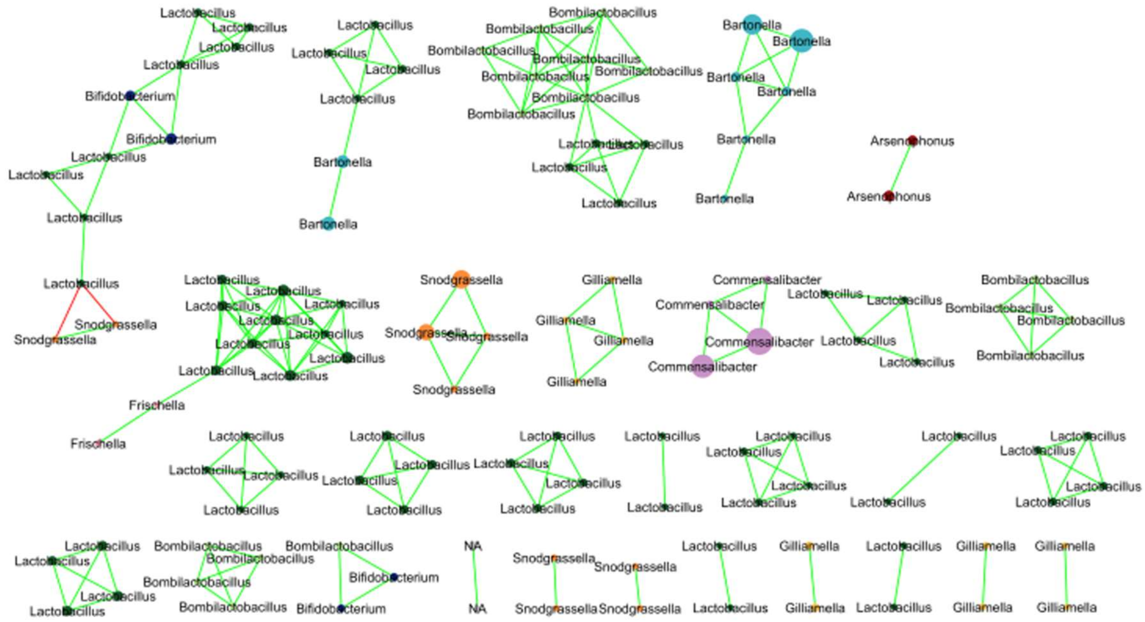

Figure S9d. Network interaction of the bees sampled from the Fumagiline/Bactocell® group at t0

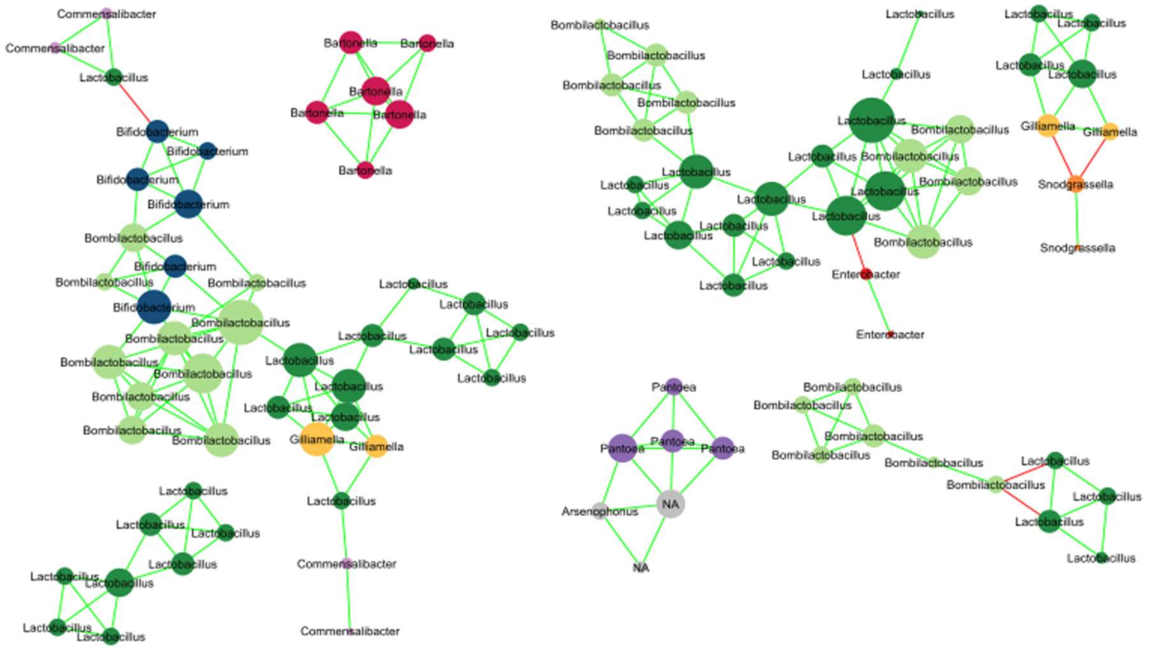

Figure S10a. Network interaction of the bees sampled from the control group at t21

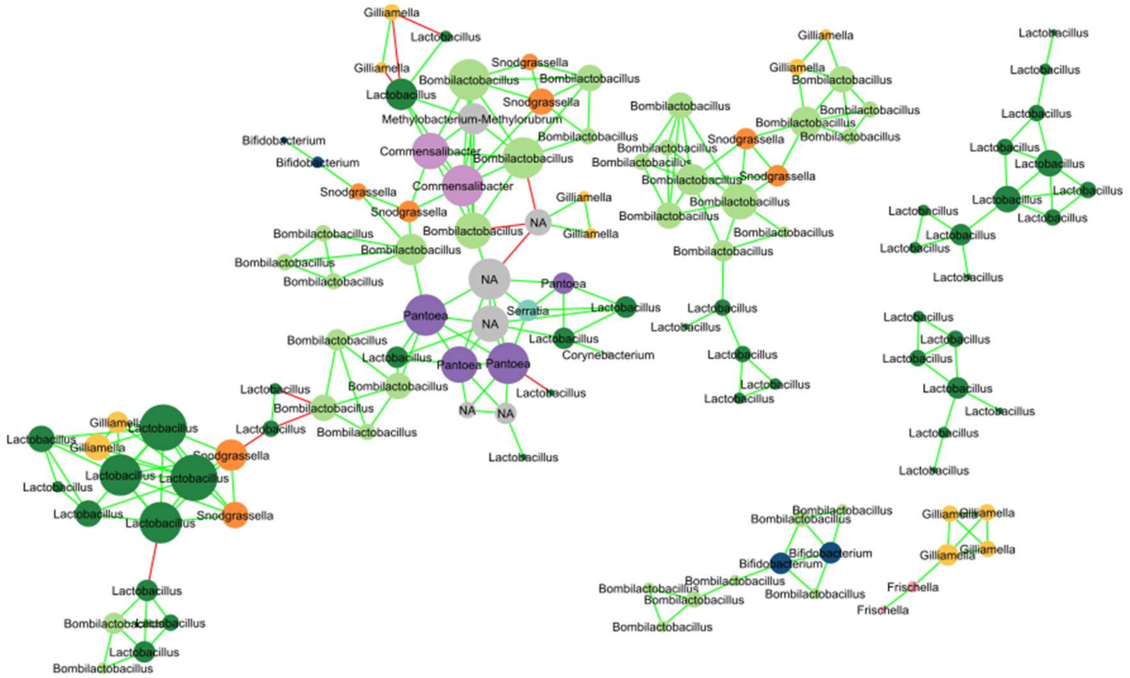

Figure S10b. Network interaction of the bees sampled from the Bactocell group at t21

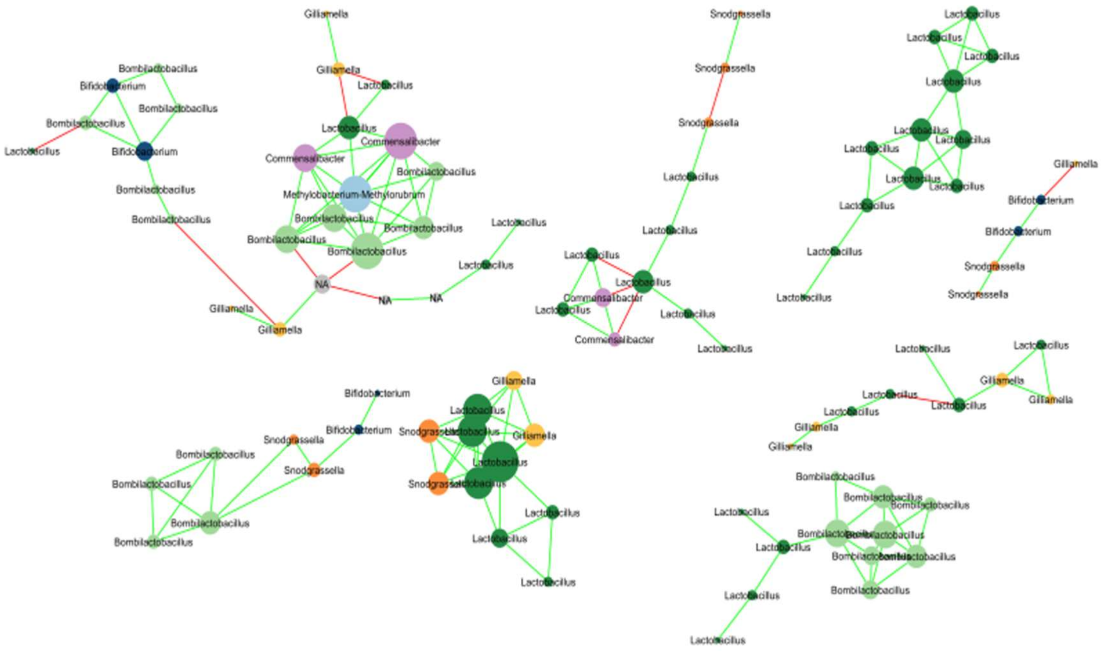

Figure S10c. Network interaction of the bees sampled from the Fumagiline group at t21

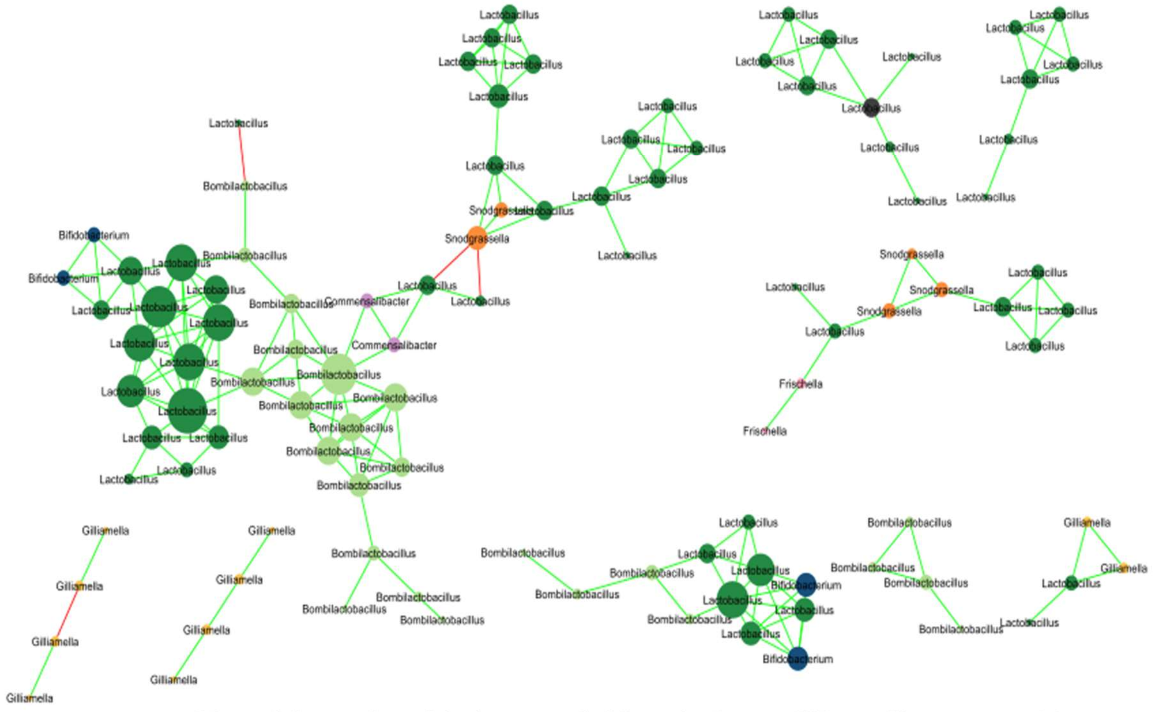

Figure S10d. Network interaction of the bees sampled from the Bactocell/Fumagiline group at t21

Group	Copresence	Mutual_exclusion	Total_interactions	Negative_interactions_rate
**Control t0 1**	634	263	897	29.319
**Control t0 2**	568	301	869	34.638
**Control t0 3**	655	370	1025	36.098
**Bacto t0 1**	691	261	952	27.416
**Bacto t0 2**	735	310	1045	29.665
**Bacto t0 3**	617	268	885	30.282
**Fum t0 1**	541	327	868	37.673
**Fum t0 2**	636	338	974	34.702
**Fum t0 3**	525	295	820	35.976
**FB t0 1**	630	272	902	30.155
**FB t0 2**	566	297	863	34.415
**FB t0 3**	602	297	899	33.037

Parameters of interaction networks created with subsamples of hives at t0.
Table S4a.

Group	Copresence	Mutual_exclusion	Total_interactions	Negative_interactions_rate
**Control t21 1**	634	263	897	29.320
**Control t21 2**	568	301	869	34.638
**Control t21 3**	655	370	1025	36.098
**Bacto t21 1**	691	261	952	27.416
**Bacto t21 2**	735	310	1045	29.665
**Bacto t21 3**	617	268	885	30.282
**Fum t21 1**	541	327	868	37.673
**Fum t21 2**	636	338	974	34.702
**Fum t21 3**	525	295	820	35.976
**FB t21 1**	630	272	902	30.155
**FB t21 2**	566	297	863	34.415
**FB t21 3**	602	297	899	33.037

Table S4b. Parameters of interaction networks created with subsamples of hives at t21.
